# Facial Palsy Secondary to Cholesteatoma: A Case-Series of 14 Patients

**DOI:** 10.3390/audiolres13010008

**Published:** 2023-01-15

**Authors:** George Psillas, Jiannis Constantinidis

**Affiliations:** 1st Otolaryngology Department, School of Medicine, Faculty of Health Sciences, Aristotle University of Thessaloniki, AHEPA Hospital, 1, Stilponos Kyriakidi St., 546 36 Thessaloniki, Greece

**Keywords:** facial palsy, cholesteatoma, facial nerve, dehiscence, fistula, mastoidectomy, facial nerve decompression

## Abstract

Background: To evaluate patients with middle ear cholesteatoma presenting with facial palsy (FP). Material-Methods: A total of 14 subjects (10 males and 4 females), with a mean age of 42.5 years, were included in our study. The majority of patients presented with incomplete FP (House–Brackmann HB II-IV, 11 cases) and the remaining 3 patients had complete facial paralysis (HB V-VI). A canal wall down mastoidectomy was performed in all the patients, followed by partial facial nerve decompression. Results: At the one-year follow-up, eleven (78.5%) patients demonstrated satisfactory recovery to HB I-II. Facial function recovered to HB grade I-II in 9 (100%) patients who were surgically treated within one month, and in 2 (40%) patients who underwent surgery after one month. The tympanic segment of facial nerve was the most common site of involvement (8 patients). The multiple regression analysis showed that a higher preoperative HB grade combined with a gradual than sudden onset of FP more likely resulted in worse postoperative HB grade. Conclusion: Early surgical removal of cholesteatoma associated with FP is more likely to result in good facial nerve recovery (78.5% of cases), when it is performed within one month from the onset of FP. According to the literature, the tympanic segment of the facial nerve was more frequently damaged (77.7%), followed by the mastoid segment (22.9%), labyrinthine segment (11.1%), and geniculate ganglion (11.1%). Labyrinthine fistula, mainly of the lateral semicircular canal, can be expected in cases of facial nerve dehiscence. The canal wall down mastoidectomy combined with partial decompression surgery was the most preferred surgical treatment for the FP secondary to cholesteatoma.

## 1. Introduction

Facial palsy (FP) is an uncommon but recognized complication of cholesteatoma; its incidence ranges from 1% to 3.4% of cholesteatoma presentations [1,2,3]. Facial palsy secondary to cholesteatoma has several causes; osteitis, bony erosion, direct compression resulting from edema, and inflammation of the nerve by bacteria or neurotoxic substances, which may be secreted from the cholesteatoma matrix can damage the facial nerve [4,5]. It has been shown that the facial nerve fills 35 to 65% of the fallopian canal; the remaining portion is filled with extra neural blood vessels and connective tissue, without leaving any empty space [5,6]; thus, edema secondary to infection can easily affect the neural transmission [5,6]. Direct pressure on the facial nerve due to cholesteatoma has also been reported [7]; however, FP was expected only after blockage of more than 50% of facial nerve fibers [8]. In slowly evolving facial palsy, the most likely etiology is erosion of the fallopian canal with compression of the facial nerve [9].

The aim of this study was to retrospectively evaluate the clinical features and outcome of cholesteatoma associated with FP. In parallel, a review of the literature was attempted in order to demonstrate data regarding the clinical presentation of cholesteatoma, onset of FP, duration of FP until surgery, and surgical observations.

## 2. Materials and Methods

Patients suffering from FP secondary to middle ear cholesteatoma, who received surgical treatment from January 2015 to January 2022 were retrospectively recruited. Patients with congenital cholesteatoma, petrous bone cholesteatoma, and iatrogenic or idiopathic (Bell’s) palsy were excluded.

The severity of FP was graded using the House–Brackmann (HB) grading system [10] as it remains the most popular assessment tool for facial nerve in the medical community; other grading scales, such as the Sunnybrook facial grading system, have shown good correlation with the HB grading system [11]. Grades HB II to IV indicate an incomplete palsy and grades V to VI a complete paralysis. The data regarding the onset and duration of FP prior to surgery and intraoperative findings were collected from the clinical records.

In order to remove the cholesteatoma, a canal wall down mastoidectomy (CWD) was performed in all the patients, in which the posterior meatal wall was dissected. A postauricular incision was made under general anesthesia in 11 patients and an endaural incision in 3 patients according to “follow the disease” approach from front to back. In case in which the fallopian canal was uncovered, a partial facial nerve decompression was performed to the smallest possible area where the facial nerve was found compressed or edematous. The epineural sheath was not incised as it was considered to be a natural barrier to the spread of infection. Intravenous antibiotics were administered for 3 to 5 days postoperatively, combined with intravenous dexamethasone (Decadron 8 mgX3/day with schema) for the same time.

Postoperative HB grade I and II was considered as good recovery, but HB grades III to VI as poor recovery. All the patients had at least a one-year follow-up after surgical intervention.

Statistical analysis was conducted with the use of SPSS v.25.0 software (SPSS, Chicago, Illinois, United States). A *p*-value of <0,05 (Student’s *t*-test) was set as the limit of statistical significance of the tests. A multiple regression was run to predict postoperative HB grade from sudden or gradual onset of FP and HB at presentation. There was linearity as assessed by partial regression plots and a plot of studentized residuals against the predicted values. There was independence of residuals, as assessed by a Durbin–Watson statistic. There was homoscedasticity, as assessed by visual inspection of a plot of studentized residuals versus unstandardized predicted values. There was no evidence of multicollinearity, as assessed by tolerance values.

## 3. Results

Fourteen patients, 10 males and 4 females, with a mean age of 42.5 ± 7.7 years (range, 16–80 years) were evaluated. The left ear was involved in 10 patients and the right ear in 4 patients. The duration of FP prior to surgery was less than one month in 9 patients, between one month and one year in 4 patients and 18 months in one patient. In 8 cases, the FP was of sudden onset and in 6 cases was gradual.

Preoperative HB grade was III in 5 patients, grade IV in 6 patients, grade V in 1 patient, and grade VI in 2 patients. All the patients presented with hearing loss; 2 (14.2%) patients had conductive hearing loss, 8 (57.1%) patients had mixed hearing loss, and 4 (28.5%) complained of total deafness. Four patients had associated symptoms such as ear discharge, tree had vertigo, and two had also headache.

The tympanic segment of facial nerve was the most common site of involvement in 8 (57.1%), followed by the tympanic and mastoid segment both injured in 4 (28.5%) patients, and the mastoid portion alone in 2 (14.2%) patients. The facial nerve was decompressed in all cases and covered by temporal fascia. The bony wall of the lateral semicircular canal was found dehiscent in 2 (14.2%) patients after removal of the cholesteatoma and it was covered with temporal fascia in both cases.

During the follow-up, eleven (78.5%) patients demonstrated satisfactory recovery to HB I (9 patients) and HB II (2 patients). Facial function recovered to HB grade I-II in 100% (9 of 9 cases) of patients who underwent surgery within one month, and in 40% (2 of 5 cases) of patients who underwent surgery after one month, with a statistically significant difference between them (*p* < 0.001) (Table 1).

## 4. Discussion

According to our results, the majority of our patients presented with incomplete FP (11 cases, 78.5%) and the remaining 3 (21.4%) patients had complete facial paralysis (HB V and VI) due to cholesteatoma (Table 1). At a follow-up of one year, the facial recovery was good (HB I and II) in 78.5% of our patients after surgical eradication of the disease and facial decompression.

Our review revealed seven studies analyzing data and interpreting results regarding the FP complicated by middle ear cholesteatoma [1,2,3,4,5,6,12]. Based on this review studies, male and middle-aged patients were mostly involved, with a cholesteatoma usually located in the left middle ear (Table 2).

Table 3 shows the preoperative HB grades of FP and the facial recovery rates after surgery in the different studies of our review. Postoperatively, about 24% of patients still had unfavorable outcome (HB III-VI), which was comparable to 21.4% of our study; inversely, satisfactory recovery of facial function (HB I-II) was shown in a mean of 70.8% of patients [87.5% [6], 81.2% [1,4], 80% [5], 69.2% [3], 60.4% [12], 36.3% [2], respectively].

In the presence of cholesteatoma, most authors prefer a CWD procedure (Table 2) for approaching the facial nerve, but also radical mastoidectomy (RM) or modified radical mastoidectomy (MRM) were also performed; in a minority of patients, canal wall up mastoidectomy (CWU) was performed or petrosectomy in case of petrous apex cholesteatoma [2]. Regarding decompression surgery, most authors prefer opening the fallopian canal at the smallest possible area (partial decompression) until reaching the normal facial nerve without redness or edema [1,2,12]; fibrosis, thinning, or interruption of the nerve was rarely found [3,6]. However, in other studies [5,6] a facial decompression was performed along the entire length of the nerve from the geniculate ganglion to the stylomastoid foramen (near-complete decompression). Wang et al. [4] concluded that there was no statistical significance for the facial recovery between partial decompression and near-complete decompression, supporting that, as observed in some cases, extent exposure of the facial nerve has been found with minimal involvement of the nerve. Controversies exist whether an incision of the epineurium should be realized; Cawthorne [13] reported that the nerve sheath incision was not necessary for patients suffering from incomplete FP, but might be performed for patients with complete facial paralysis; other authors [1,6] did not perform nerve sheath incision even when the fallopian canal was opened.

In our series, the tympanic segment of facial nerve was the most affected site (85.7%), followed by the mastoid portion (14.2%). Cholesteatoma-induced bone destruction of the fallopian canal could be the result of a multifactorial process, involving pressure necrosis, osteolytic enzymes secretion, and osteoclastic activity [14,15]. In our review (Table 4), the tympanic segment was more frequently damaged in 77.7% (105/135) of patients; this corroborates the current understanding of the growth patterns of cholesteatoma, in which cholesteatoma closely approximates the tympanic segment of the facial nerve [16]. The mastoid segment (22.9%, 31/135), followed by the labyrinthine segment (11.1%, 15/135) and geniculate ganglion (11.1%, 15/135) was found at the time of surgery to be more commonly damaged.

In Li et al.’s study [12], 8 (16.6%) out of 48 patients and with FP secondary to cholesteatoma had intact fallopian canal and had complete recovery after facial exploration. Similarly, Yetiser et al. [6] also showed 4 (25%) out of 16 patients with no destruction of the facial canal. Even though fallopian canal dehiscence has been described in normal population [17], it is more commonly found during cholesteatoma surgery [14] and may be a negative prognostic indicator for the outcome [12]. The incidence of facial nerve dehiscence varies considerably at the time of cholesteatoma surgery and ranges from 8.9% to 45.5% [14]. It has been reported that around 80% of patients have a dehiscence of the tympanic segment of facial nerve because it is covered by very thin layer of bone [15,18]; this incidence was found higher in Ikeda’s [1] (98%) and Lin’s [19] (92%) studies, making this area more vulnerable for facial nerve injury. The facial nerve could also be exposed at the geniculate ganglion and labyrinthine segment, mainly with the extension of either a petrous apex cholesteatoma [2] or an anterior epitympanic cholesteatoma [9]. In particular, Ozkul et al. [5] have shown that, during decompression surgery in the fallopian canal, when the tympanic segment was exposed, the mastoid segment was also involved in the half of cases. Dehiscence of the fallopian canal is a real concern for the otologist due to the risk of injury to the facial nerve. Therefore, in order to obtain a more secure perioperative evaluation of the facial nerve, both microscopy and palpation should be undertaken to ensure the presence or absence of dehiscence [14,18].

Labyrinthine fistulae are likely to occur when a facial nerve dehiscence is present, probably due to the destructive effect of cholesteatoma on the bone covering the semicircular canal. Although the incidence of labyrinthine fistula associated with cholesteatoma is about 4% to 15% [20], the incidence is increased to a range from 12.8% to 15.4% [14,15,18] when a facial nerve dehiscence is present; Moody et al. [15] quoted an approximate three-fold higher incidence of fistula in those ears with a facial nerve dehiscence, Gulotta et al. [18] five-fold and Trinidade and Yang [14] seven-fold, respectively. Moreover, labyrinthine fistula was more commonly found in the lateral semicircular canal (50%), superior semicircular canal (38%), posterior semicircular canal (13%), and vestibule (25%) [1]. These findings should alert the operating otologist, during removal of cholesteatoma, to the high likelihood of labyrinthine fistula and facial nerve dehiscence coexisting.

Early surgical removal of cholesteatoma and decompression contribute to better outcome of facial nerve (Table 5). A prolonged infection of the nerve fibers may lead to irreversible damage of facial nerve and the recovery of the recovery of facial function depended on the prompt removal of the infection [5]; similar to Wang et al.’s study [4], the majority of our patients underwent surgical intervention within one month with good recovery of the facial nerve.

In the Quaranta et al. [3]’s series, all the patients operated on within 7 days from the onset of facial palsy had complete recovery (grade HB I), whereas patients operated on 7 days or more showed a variable outcome. Similarly, Ozkul et al. [5] suggested that, regardless of the degree of facial palsy, onset of facial palsy, age, and any previous otological surgery, the patients should operate on within 15 days from facial palsy. Li et al. [12] showed that 81% of cases with duration of facial palsy ≤2 months achieved good recovery, while only 21.1% of their patients with duration of facial palsy >2 months attained good recovery (*p* < 0.01). Siddiq et al. [2] showed that facial nerve recovery could be anticipated if it has been present for up to 7 months, but not if facial palsy has been present for more than 3½ years; however, in their series, eight out of 11 patients had petrous apex cholesteatoma associated with facial palsy and only 3 were confined to middle-ear cleft.

The multiple regression analysis was performed in three [1,2,6] out of the seven studies, as the data from the remaining studies were not sufficient. Our statistical results showed that a higher preoperative HB grade combined with a gradual than sudden onset of FP more likely resulted in worse postoperative HB grade (Table 6). The multiple regression model statistically significantly predicted in the Ikeda et al.’s [1] study, in which that there was a significant relationship between HB at presentation with postoperative HB grade (*p* < 0.05) and sudden/gradual onset of FP with postoperative HB grade (*p* < 0.01). Specifically, a 0.508 increase (±0.225) was found in the outcome of postoperative HB grade for every 1 unit of increase in HB at presentation; in addition, the predicted postoperative HB grade for patients with gradual onset of FP was 2.362 greater than predicted for patients with sudden onset of FP. Statistically significant relationship between HB at presentation with the outcome was also noted in the Siddiq et al.’s [2] and Yetiser et al.’s [6] study, respectively (*p* < 0.01 for each study). Similarly, Quaranta et al. [3] found that almost all the patients with lower preoperative HB grades (grades II and III) had good recovery compared to only 50% of patients with higher preoperative HB (grades IV-VI), but this difference was not significant (*p* = 0.266). It is possible that the above finding was based on the different pathophysiologic mechanisms regarding the facial recovery, in which the sudden onset of FP was due to the secretion of neurotoxic substances from the cholesteatoma, but the gradual FP was caused by more extensive damage, associated with the erosion of the fallopian canal and compression of the facial nerve [9].

The use of electrophysiological testing for the evaluation of facial nerve function was limited in these studies; Ikeda et al. [1] reported that the preoperative results of NET (nerve excitability testing) were abnormal in 3 patients, all of whom had severe FP, and normal in the remaining 13. Regarding ENoG (electroneurography of facial nerve), Wang et al. [4] demonstrated that the facial nerve function recovered to HB grade II in 87.5% (14 cases) of patients in whom ENoG showed lighter functional damage of facial nerve and in 75% (12 cases) of patients in whom ENoG showed more severe damage, with no significant difference between them (*p* > 0.05).

## 5. Conclusions

Early surgical removal of cholesteatoma associated with FP is more likely to result in good facial nerve recovery (78.5% of cases), when it is performed within one month from the onset of FP. The tympanic segment was more frequently damaged by the cholesteatoma (85.7 in our study vs. 77.7% according to our review), followed by the mastoid segment. Labyrinthine fistula, mainly of the lateral semicircular canal, can be expected in cases of facial nerve dehiscence, due to the destructive effect of the cholesteatoma. Based on the literature data, a higher preoperative HB grade combined with a gradual than sudden onset of FP more likely resulted in worse postoperative HB grade; moreover, the canal wall down mastoidectomy combined with partial decompression surgery was the most preferred surgical treatment for the FP secondary to cholesteatoma.

## Figures and Tables

**Table 1:** Facial function before and after removal of cholesteatoma and facial decompression surgery (according to House–Brackmann grading system). ** *p* < 0.01, *** *p* < 0.001.

	Preop.HB Grade				Postop.HB Grade				
Duration	HB III	IV	V	VI	HB I	II	III	IV	Total
1 month	3 (21.4%)	4 (28.5%)	1 (7.1%)	1 (7.1%)	7 (50%)	2 (14.2%)	0	0	9 (64.2%) ***
>1 month	2 (14.2%)	2 (14.2%)	0	1 (7.1%)	2 (14.2%)	0	2 (14.2%)	1 (7.1%)	5 (35.5%) **

**Table 2:** Studies including number (Nb) and characteristics of patients, onset of facial palsy (FP), surgical technique for removal of cholesteatoma and postoperative follow-up. CWU: canal wall up mastoidectomy, CWD: canal wall down mastoidectomy, RM: radical mastoidectomy, MRM: modified radical mastoidectomy, m = month, y = year.

	Nb of Patients, R/L Side	Mean Age(y) ± sd, Gender (M/F)	Onset of FP (Sudden/Gradual)	Surgical Technique (nb of Patients)	Follow-Up
Ikeda et al. [1]	16, -	46.4 ± 20.2 11/5	12/4	CWD (16)	At least 6 m
Siddiq et al. [2]	11, -	56 7/4	8/3	MRM (3) Total petrosectomy (8)	1 m–5 y
Quaranta et al. [3]	13, 5/8	50.2 9/4	11/2	CWU (2) CWD (4) RM (7)	1 y–26 y
Wang et al. [4]	32, 14/18	45.4 ± 5.4 22/10	-	MRM (32)	At least 1 y
Ozkul et al. [5]	15, -	58.4 ± 8.3 8/7	-	CWU (1) CWD (6) RM (8)	1 y–174 m
Yetiser et al. [6]	16, 7/9	32.1 ± 19.3 12/4	3/13	CWD (12) RM or MRM (3) CWD + petrosectomy (1)	-
Li et al. [12]	48, -	53.2 ± 7.5 28/20	-	CWD (40)	At least 1 y
Total	151, 26/35	48.8, 97/54	34/22	CWD (79) RM or MRM (53) CWU (3) Petrosectomy (9)	

**Table 3:** Number of patients relating to House–Brackmann grade before and after surgery for cholesteatoma associated with facial palsy. NS: not specified.

Preop.HB Grade	Ikeda et al. [1]	Siddiq et al. [2]	Quaranta et al. [3]	Wang et al. [4]	Ozkul et al. [5]	Yetiser et al. [6]	Li et al. [12]	Total
II	1	1	2	-	2		NS	6/87, 6.9%
III	8	3	3	16	4		NS	34/87, 39%
IV	2	2	2	14	7	II–IV (11, 68.7%)	NS	27/87, 31%
V	3	1	2	2	1		NS	9/87, 10.3%
VI	2	4	4	-	1	V–VI (5, 31.2%)	NS	11/87, 12.6%
Postop.HB grade								
I	11	1	7		11	8		
II	2	3	2	I–II = 26	1	6	I–II = 29	I + II 107/151, 70.8%
III	1	2	2	4	2	2		13/103, 12.6%
IV	-	-	-	2	-	-		4/103, 3.8%
V	-	1	-	-	-	-		1/103, 0.9%
VI	2	4	-	-	1	-	III–VI = 19	7/103, 6.8%

**Table 4:** Segment(s) of facial nerve damaged from cholesteatoma in different studies (total number of patients = 135). L = labyrinthine, G = geniculate ganglion, T = tympanic, M = mastoid.

	L	G	T	M	LG	LT	GT	TM	LGM	LTM	LGTM
Ikeda et al. [1]	5	6	15	5							
Siddiq et al. [2]	1		4		3		1		1		1
Quaranta et al. [3]			5	1		2		4		1	
Wang et al. [4]			18								
Ozkul et al. [5]			14	1							
Li et al. [12]	1		30	10			3	7			
Total	7	6	86	17	3	2	4	11	1	1	1

**Table 5:** Number of patients in different studies according to duration time of facial palsy until surgery (removal of cholesteatoma and facial decompression), NS: not specified.

Duration	Ikeda et al. [1]	Siddiq et al. [2]	Quaranta et al. [3]	Wang et al. [4]	Ozkul et al. [5]	Yetiser et al. [6]	Li et al. [12]	Total
1 month	10	2	9	20	15	16	NS	72/103, 69.9%
1 month–1 year	4	2	4	NS	-	-	NS	10/71, 14%
>1 year	2	7	-	NS	-	-	NS	9/71, 12.6%

**Table 6:** Multiple regression analysis to predict facial recovery (postoperative HB grade) from sudden/gradual onset of facial palsy and HB grade at presentation. B: unstandardized regression coefficient, CI: confidence interval, LL: lower limit, UL: upper limit, SE B: standard error of the coefficient, ΔR^2^: adjusted R^2^, * *p* < 0.05, ** *p* < 0.01, *** *p* < 0.001.

Authors			95% CI for B			
		B	LL	UL	SE B	ΔR^2^
Ikeda et al. [1]						0.741 ***
	HB at presentation	0.508 *	0.022	0.994	0.225	
	Sudden/ gradual onset	2.362 **	1.033	3.691	0.615	
Siddiq et al. [2]						0.837 ***
	HB at presentation	1.145 **	0.674	1.615	0.204	
	Sudden/ gradual onset	0.491	−1.022	2.003	0.656	
Yetiser et al. [6]						0.497 **
	HB at presentation	0.569 **	0.250	0.888	0.148	
	Sudden/ gradual onset	0.034	−0.724	0.793	0.351	

## Data Availability

The data presented in this study is available upon request from the corresponding author.
